# Supplementary figures and images for: Evolving patterns of COVID-19 mortality in US counties: A longitudinal study of healthcare, socioeconomic, and vaccination associations
Source: PLOS Glob Public Health. 2024 Sep 10;4(9):e0003590. doi: 10.1371/journal.pgph.0003590 (PMC11386416; doi:10.1371/journal.pgph.0003590)

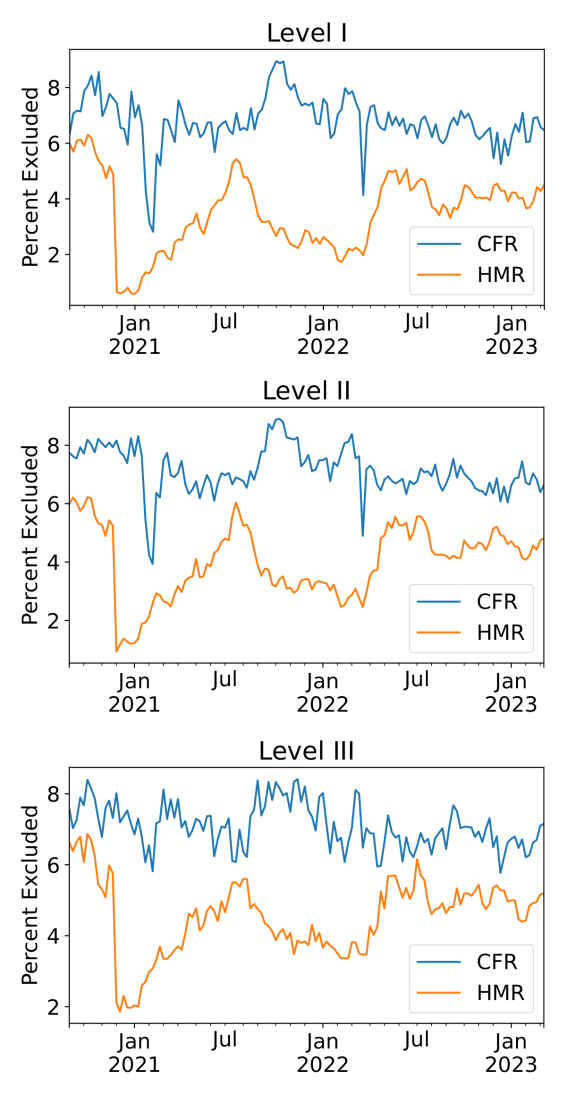

Supplement: S1 Fig — Percentage of excluded counties in each run of the analyses using the three times the mean of Cook’s distance threshold. (TIFF) [file pgph.0003590.s001.tiff]

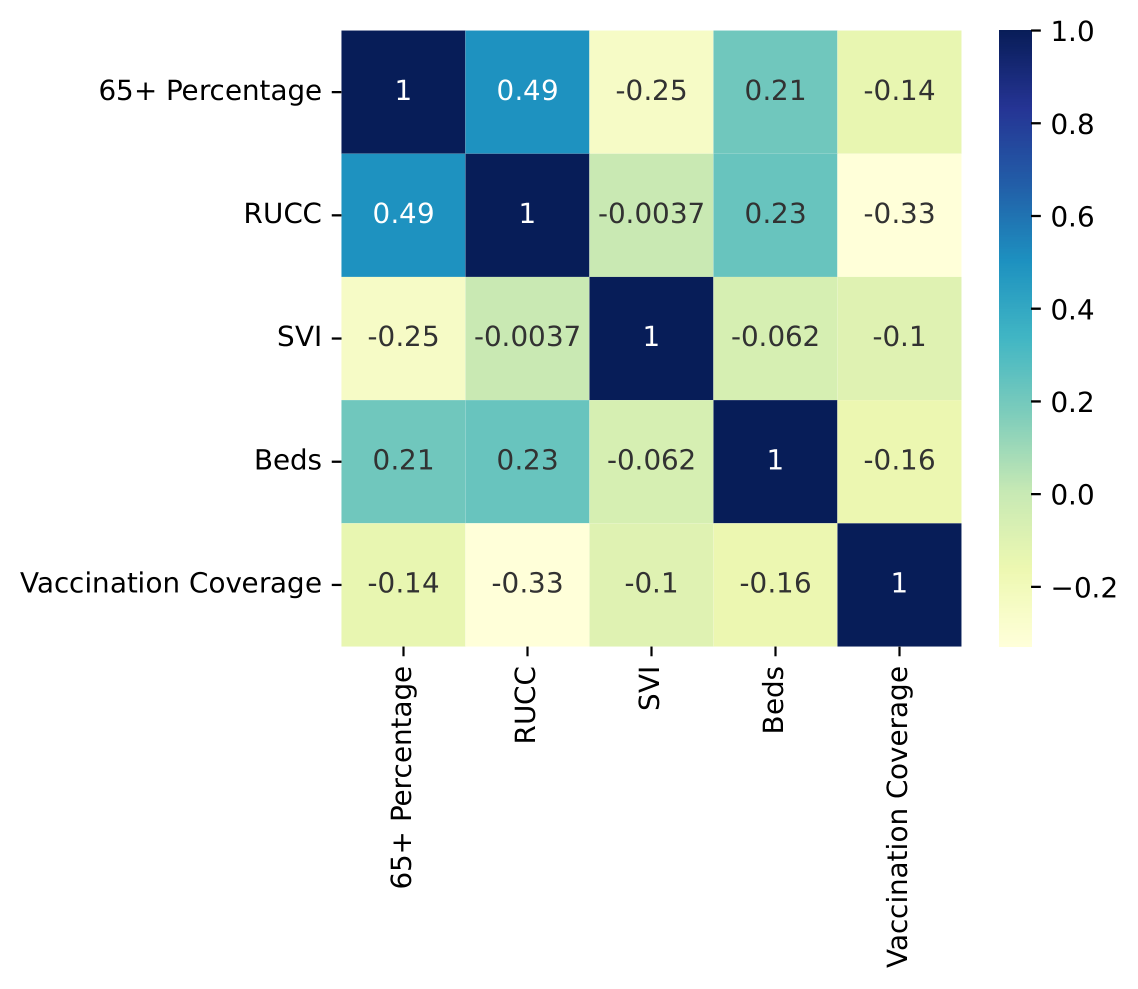

Supplement: S2 Fig — Pearson correlation coefficients among independent variables for the level I analysis. (TIF) [file pgph.0003590.s002.tif]

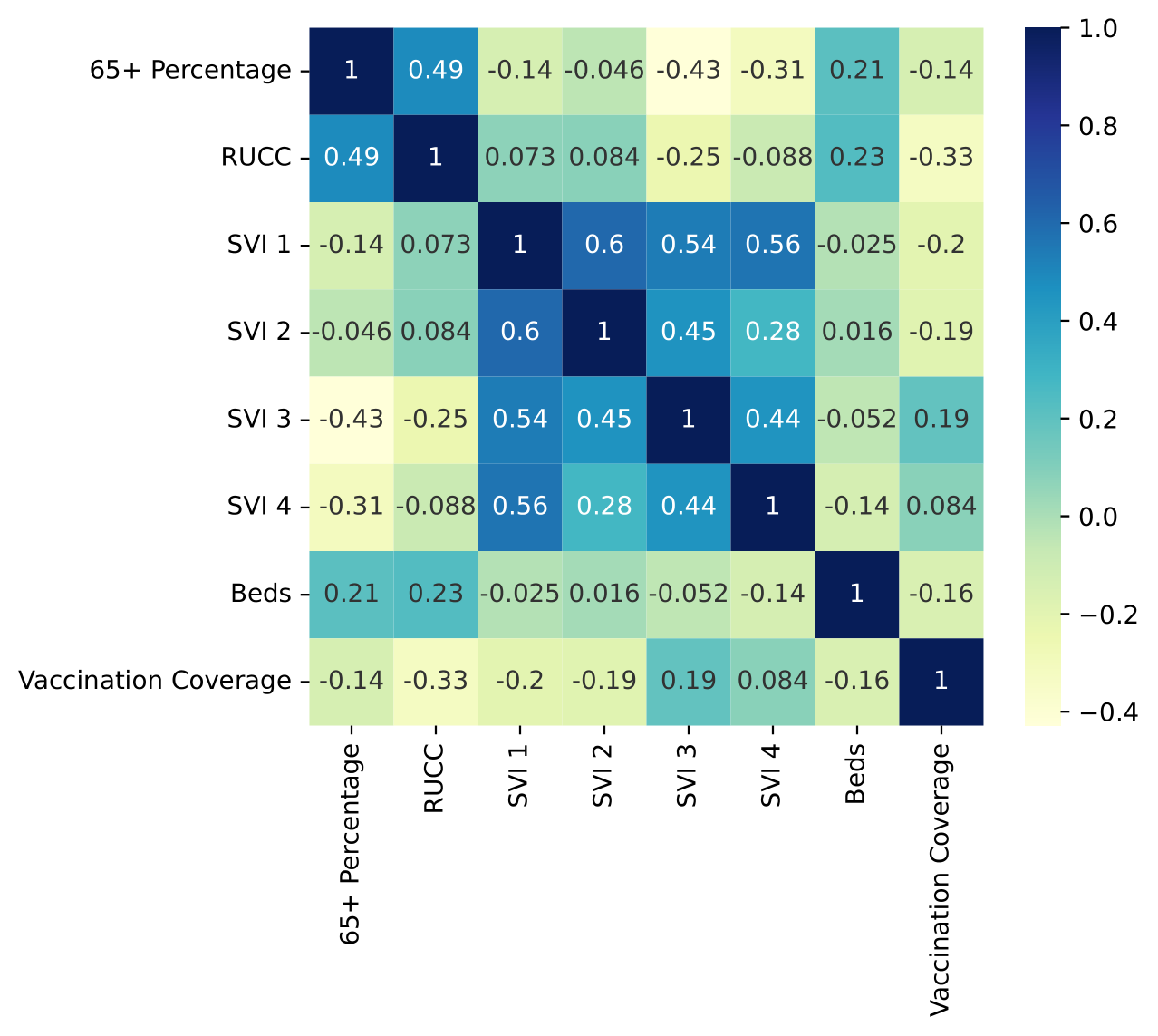

Supplement: S3 Fig — Pearson correlation coefficients among independent variables for the level II analysis. (TIF) [file pgph.0003590.s003.tif]

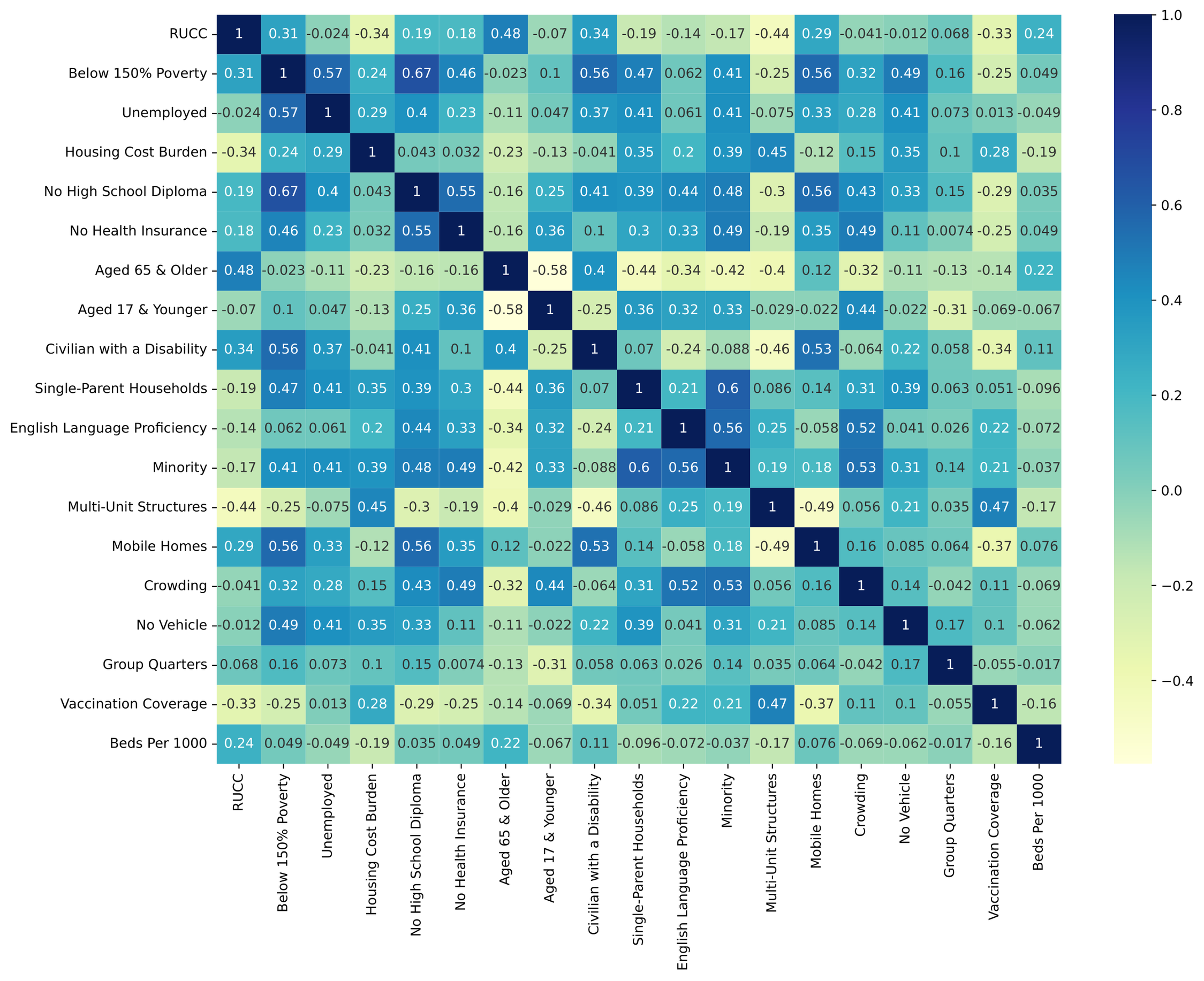

Supplement: S4 Fig — Pearson correlation coefficients among independent variables for the level III analysis. (TIF) [file pgph.0003590.s004.tif]

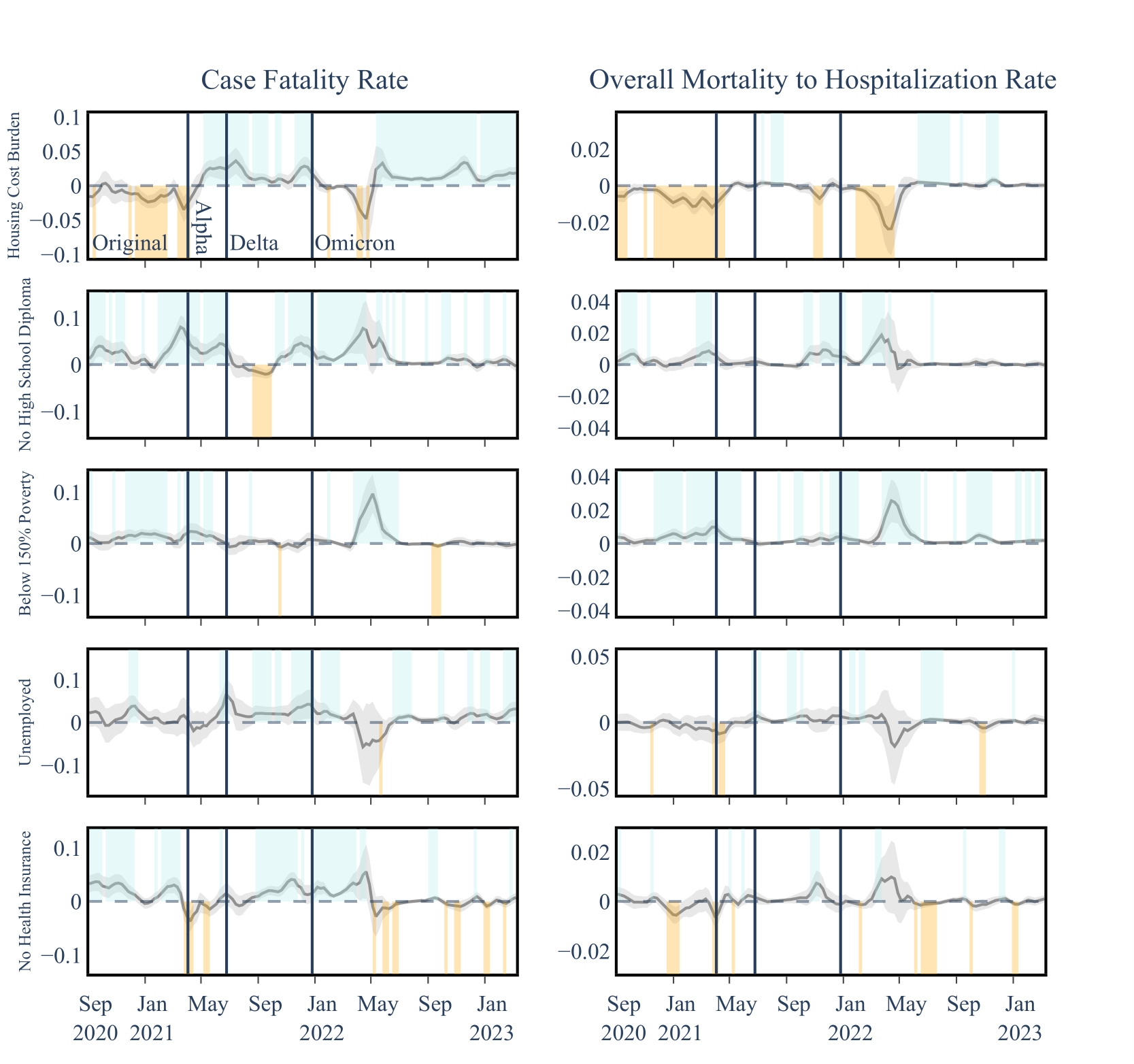

Supplement: S5 Fig — SVI theme I is almost consistently positively associated with mortality measures and education and poverty amplify this association but the other measures provide less consistent relationships. (TIF) [file pgph.0003590.s005.tif]

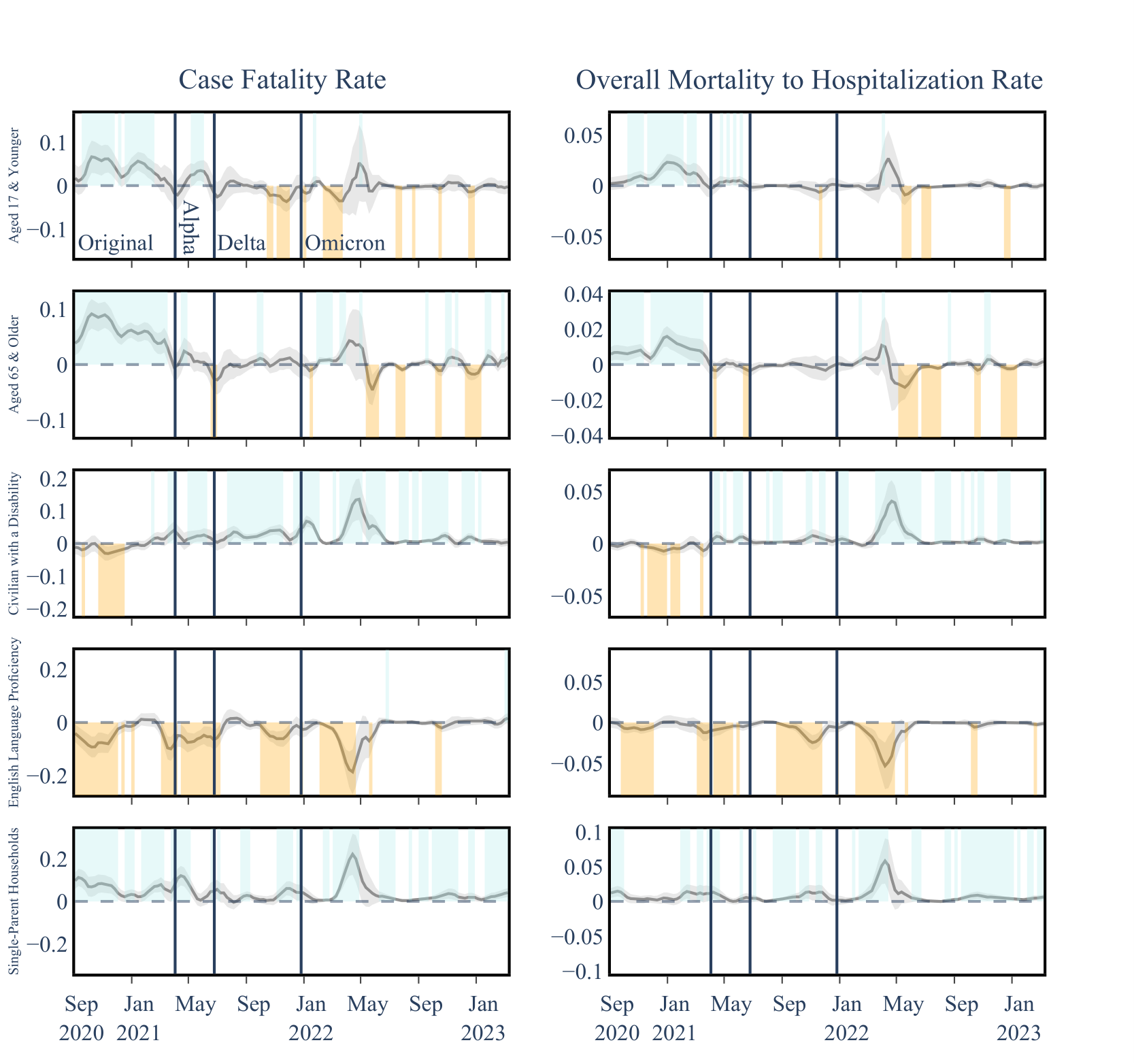

Supplement: S6 Fig — Single-parent households show a consistent positive association with both CFR and OMHR. (TIF) [file pgph.0003590.s006.tif]

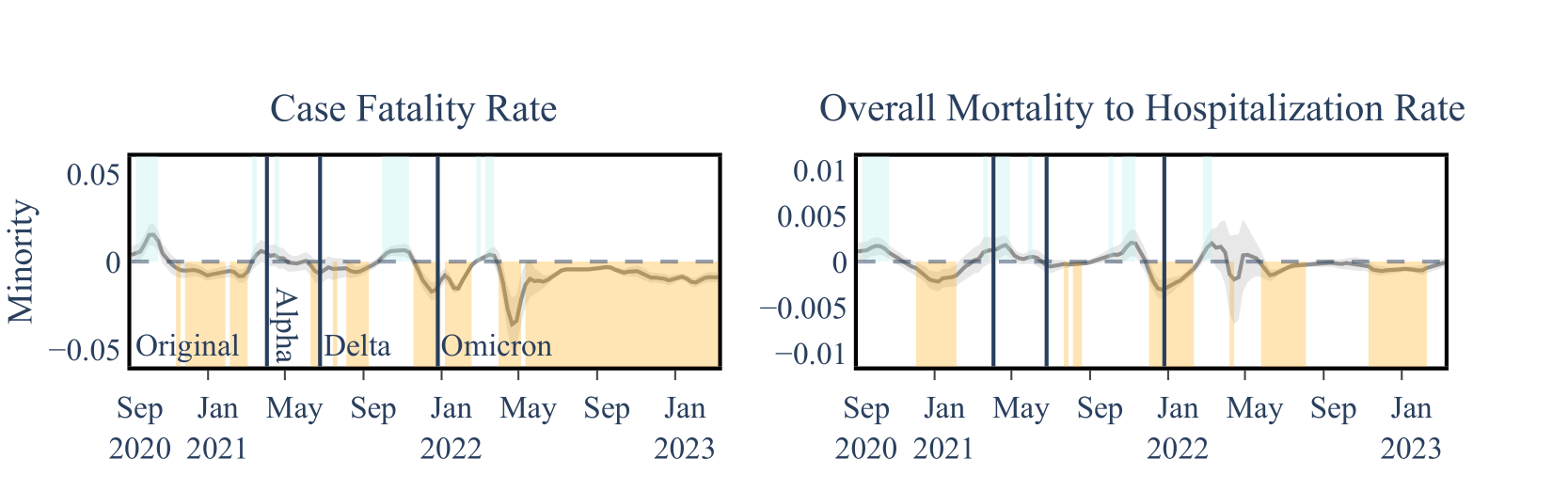

Supplement: S7 Fig — (TIF) [file pgph.0003590.s007.tif]

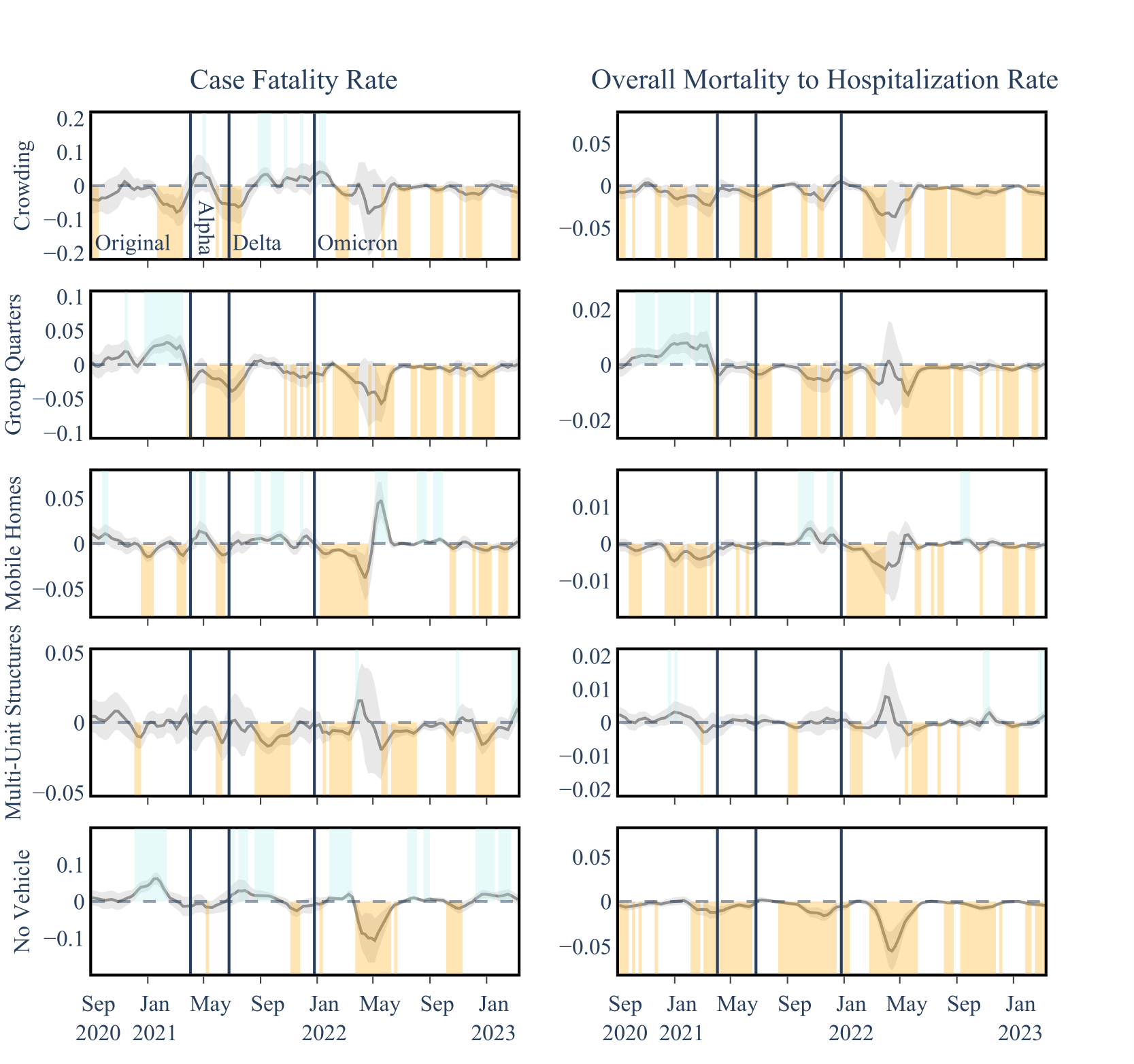

Supplement: S8 Fig — The housing and transportation variables exhibited mixed patterns, predominantly inconsistent negative associations with COVID-19 mortality outcomes. (TIF) [file pgph.0003590.s008.tif]

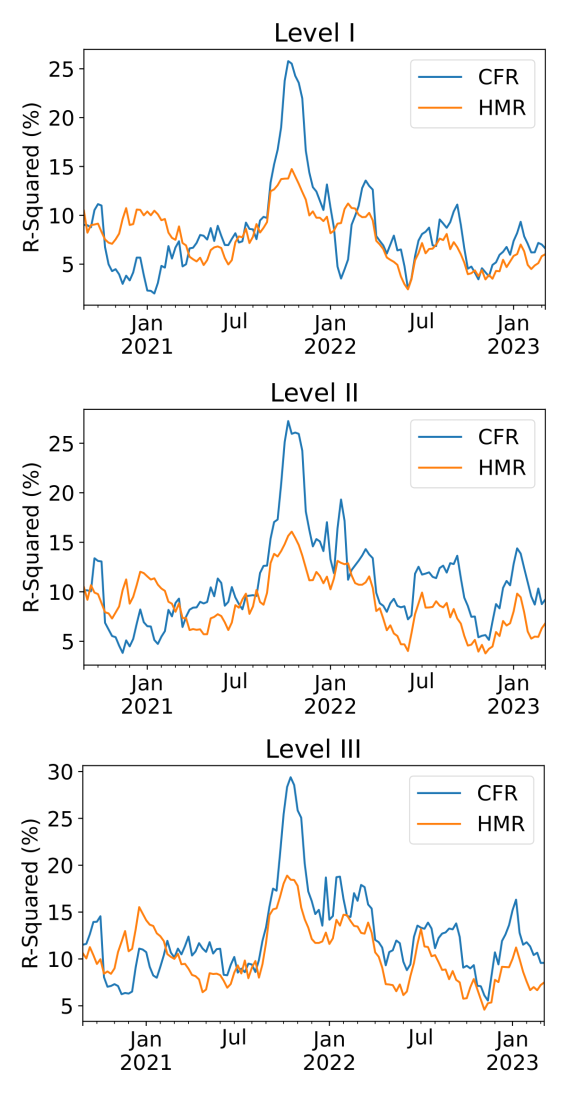

Supplement: S9 Fig — (TIFF) [file pgph.0003590.s009.tiff]

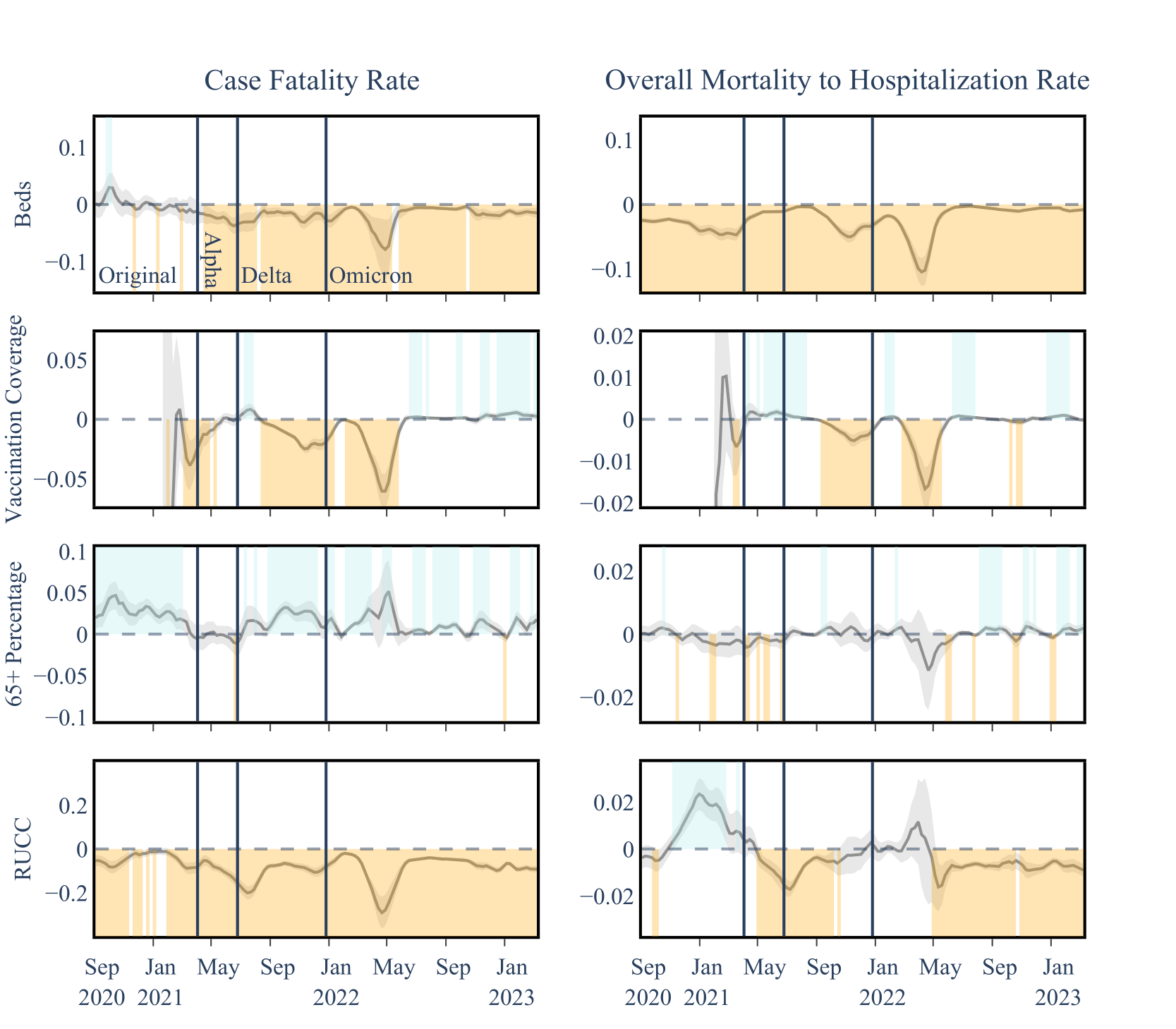

Supplement: S10 Fig — The associations here are primarily similar to those of Level I analysis (see Fig 5), with lower levels of significance in some cases. The 65+ Age Group specifically exhibits more frequent insignificant periods, which is expected as SVI theme 2 has it as one of its constituent variables. Vaccination coverage also exhibits more positive periods concerning CFR in the late Omicron phase compared to Level I analyses. SVI variables for this analysis are presented in the main body of the paper, see Fig 7. (TIF) [file pgph.0003590.s010.tif]
