# Supplementary material for: Evolving patterns of COVID-19 mortality in US counties: A longitudinal study of healthcare, socioeconomic, and vaccination associations
Source: PLOS Glob Public Health. 2024 Sep 10;4(9):e0003590. doi: 10.1371/journal.pgph.0003590 (PMC11386416; doi:10.1371/journal.pgph.0003590)
Supplement: S1 Table — For each variable and variant period, the overall association, the week % of statistical significance, the coefficients, and the p-values are provided. Those with statistical significance during more than half of the weeks are highlighted in bold. (PDF) [file pgph.0003590.s011.pdf]

S1 Table. Summary of key results for CFR with SVI Level III variables. For each variable and variant period, the overall association, the week % of statistical significance, the coefficients, and the p-values are provided. Those with statistical significance during more than half of the weeks are highlighted in bold.

	**Variable**	**Variant**	**Assoc.**	**Weeks significant**			**Coeffs. avg (std)**		**P-value* avg (std)**	
				(%)-	(%)+	Total	-	+	-	+
**SVI 1: Socioeconomic Status**	Below 150% Poverty	Original	**Pos.**	0	**55**	31		0.0167 (0.0041)		0.01 (0.02)
		Alpha	**Pos.**	0	**58**	12		0.0202 (0.0050)		7e-03 (6e-03)
		Delta	Insig.	4	4	26	-0.0128 (nan)	0.0089 (nan)	9e-04 (nan)	0.03 (nan)
		Omicron	Insig.	5	24	63	-0.0052 (0.0017)	0.0406 (0.0347)	0.01 (0.01)	5e-03 (8e-03)
	Unemployed	Original	Insig.	0	10	31		0.0388 (0.0110)		3e-03 (4e-03)
		Alpha	Insig.	0	17	12		0.0515 (0.0007)		8e-03 (1e-03)
		Delta	**Pos.**	0	**58**	26		0.0338 (0.0200)		0.02 (0.01)
		Omicron	Insig.	2	38	63	-0.0557 (nan)	0.0201 (0.0086)	0.02 (nan)	4e-03 (7e-03)
	Housing Cost Burden	Original	**Neg.**	**52**	0	31	-0.0213 (0.0084)		0.01 (0.01)	
		Alpha	**Pos.**	8	**58**	12	-0.0190 (nan)	0.0255 (0.0038)	0.04 (nan)	4e-03 (7e-03)
		Delta	**Pos.**	0	**73**	26		0.0206 (0.0100)		0.01 (0.02)
		Omicron	**Pos.**	6	**67**	63	-0.0370 (0.0214)	0.0162 (0.0085)	0.02 (0.01)	2e-03 (7e-03)
	No High-School Diploma	Original	**Pos.**	0	**61**	31		0.0403 (0.0210)		4e-03 (7e-03)
		Alpha	**Pos.**	0	**92**	12		0.0365 (0.0071)		4e-03 (9e-03)
		Delta	Insig.	23	42	26	-0.0184 (0.0030)	0.0330 (0.0135)	2e-03 (2e-03)	7e-03 (1e-02)
		Omicron	Insig.	0	44	63		0.0265 (0.0241)		0.01 (0.01)
	No Health Insurance	Original	**Pos.**	3	**68**	31	-0.0485 (nan)	0.0278 (0.0084)	1e-07 (nan)	5e-03 (1e-02)
		Alpha	Insig.	33	8	12	-0.0249 (0.0099)	0.0218 (nan)	0.01 (0.01)	0.05 (nan)
		Delta	**Pos.**	0	**54**	26		0.0256 (0.0111)		2e-03 (7e-03)
		Omicron	Insig.	17	33	63	-0.0133 (0.0111)	0.0233 (0.0180)	0.02 (0.02)	5e-03 (1e-02)
**SVI 2: Household Characteristics**	Aged 65 & Older	Original	**Pos.**	0	**94**	31		0.0618 (0.0189)		2e-04 (7e-04)
		Alpha	Insig.	8	17	12	-0.0293 (nan)	0.0323 (0.0067)	0.02 (nan)	0.01 (0.02)
		Delta	Insig.	4	8	26	-0.0361 (nan)	0.0135 (0.0023)	7e-03 (nan)	0.03 (0.02)
		Omicron	Insig.	24	24	63	-0.0207 (0.0152)	0.0187 (0.0130)	9e-03 (2e-02)	0.02 (0.02)
	Aged 17 & Younger	Original	**Pos.**	0	**65**	31		0.0499 (0.0152)		6e-03 (1e-02)
		Alpha	Insig.	0	33	12		0.0359 (0.0077)		5e-03 (8e-03)
		Delta	Insig.	23	0	26	-0.0315 (0.0081)		0.01 (0.02)	
		Omicron	Insig.	21	3	63	-0.0179 (0.0118)	0.0599 (0.0493)	0.02 (0.02)	5e-03 (7e-03)
	Civilian with a Disability	Original	Insig.	29	10	31	-0.0249 (0.0054)	0.0346 (0.0121)	0.02 (0.02)	5e-03 (3e-03)
		Alpha	**Pos.**	0	**58**	12		0.0269 (0.0083)		0.02 (0.02)
		Delta	**Pos.**	0	**73**	26		0.0306 (0.0093)		2e-03 (4e-03)
		Omicron	**Pos.**	0	**60**	63		0.0390 (0.0394)		6e-03 (1e-02)
	Single-Parent Households	Original	**Pos.**	0	**81**	31		0.0697 (0.0273)		4e-03 (7e-03)
		Alpha	**Pos.**	0	**50**	12		0.0994 (0.0270)		2e-04 (5e-04)
		Delta	Insig.	0	38	26		0.0470 (0.0157)		7e-03 (8e-03)
		Omicron	**Pos.**	0	**59**	63		0.0489 (0.0610)		0.01 (0.01)
	English Language Proficiency	Original	**Neg.**	**65**	0	31	-0.0672 (0.0296)		8e-03 (1e-02)	
		Alpha	**Neg.**	**92**	0	12	-0.0614 (0.0125)		6e-03 (1e-02)	
		Delta	Insig.	42	0	26	-0.0520 (0.0156)		7e-03 (1e-02)	
		Omicron	Insig.	24	3	63	-0.0768 (0.0690)	0.0143 (0.0087)	3e-03 (6e-03)	0.03 (0.01)
**SVI 3**	Racial & Ethnic Minority Status	Original	Insig.	45	23	31	-0.0067 (0.0017)	0.0107 (0.0055)	3e-03 (4e-03)	9e-03 (1e-02)
		Alpha	Insig.	17	8	12	-0.0076 (0.0005)	0.0068 (nan)	0.02 (0.01)	0.03 (nan)
		Delta	Insig.	42	23	26	-0.0092 (0.0053)	0.0063 (0.0006)	3e-03 (8e-03)	4e-04 (6e-04)
		Omicron	**Neg.**	**87**	5	63	-0.0097 (0.0076)	0.0040 (0.0019)	1e-03 (5e-03)	0.01 (0.01)

Continued: Summary of results for CFR - Level III

	Variable	Variant	Assoc.	Weeks significant			Coeffs. avg (std)		P-value* avg (std)	
				(%)-	(%)+	Total	-	+	-	+
SVI 4: Housing Type & Transportation	Multi-Unit Structures	Original	Insig.	6	0	31	-0.0120 (0.0011)		5e-03 (4e-03)	
		Alpha	Insig.	17	0	12	-0.0160 (0.0030)		0.01 (0.01)	
		Delta	Insig.	42	0	26	-0.0122 (0.0034)		7e-03 (1e-02)	
		Omicron	Insig.	43	6	63	-0.0092 (0.0037)	0.0130 (0.0047)	7e-03 (1e-02)	0.03 (0.01)
	Mobile Homes	Original	Insig.	23	10	31	-0.0132 (0.0032)	0.0135 (0.0025)	0.01 (0.02)	0.02 (0.02)
		Alpha	Insig.	25	25	12	-0.0125 (0.0035)	0.0147 (0.0022)	0.02 (0.03)	0.01 (0.01)
		Delta	Insig.	0	27	26		0.0084 (0.0016)		6e-03 (8e-03)
		Omicron	Insig.	40	16	63	-0.0124 (0.0103)	0.0176 (0.0216)	6e-03 (1e-02)	7e-03 (1e-02)
	Crowding	Original	Insig.	32	0	31	-0.0607 (0.0153)		8e-03 (1e-02)	
		Alpha	Insig.	25	8	12	-0.0575 (0.0011)	0.0479 (nan)	0.02 (0.01)	0.04 (nan)
		Delta	Insig.	15	23	26	-0.0612 (0.0099)	0.0333 (0.0080)	0.02 (0.01)	0.02 (0.02)
		Omicron	Insig.	33	5	63	-0.0215 (0.0121)	0.0489 (0.0113)	0.02 (0.02)	0.01 (0.02)
	No Vehicle	Original	Insig.	0	32	31		0.0443 (0.0155)		2e-03 (5e-03)
		Alpha	Insig.	8	0	12	-0.0198 (nan)		0.03 (nan)	
		Delta	Insig.	12	38	26	-0.0254 (0.0051)	0.0217 (0.0082)	1e-02 (1e-02)	0.02 (0.02)
		Omicron	Mixed	29	35	63	-0.0515 (0.0388)	0.0142 (0.0066)	0.01 (0.02)	8e-03 (1e-02)
	Group Quarters	Original	Insig.	3	42	31	-0.0257 (nan)	0.0272 (0.0056)	0.01 (nan)	1e-03 (2e-03)
		Alpha	**Neg.**	**67**	0	12	-0.0257 (0.0076)		3e-03 (3e-03)	
		Delta	Insig.	38	0	26	-0.0249 (0.0089)		0.01 (0.02)	
		Omicron	**Neg.**	**57**	0	63	-0.0168 (0.0164)		0.01 (0.02)	
non-SVI variables	Beds	Original	Insig.	42	0	31	-0.0213 (0.0062)		0.01 (0.01)	
		Alpha	**Neg.**	**100**	0	12	-0.0281 (0.0092)		4e-03 (1e-02)	
		Delta	**Neg.**	**96**	0	26	-0.0222 (0.0086)		3e-03 (7e-03)	
		Omicron	**Neg.**	**86**	0	63	-0.0189 (0.0188)		3e-03 (6e-03)	
	Vaccination Coverage	Original	Insig.	36	9	11	-0.0472 (0.0048)	0.0508 (nan)	2e-05 (2e-05)	0.03 (nan)
		Alpha	**Neg.**	**58**	0	12	-0.0141 (0.0058)		7e-03 (1e-02)	
		Delta	**Neg.**	**73**	4	26	-0.0138 (0.0068)	0.0065 (nan)	7e-05 (3e-04)	0.04 (nan)
		Omicron	Mixed	27	32	63	-0.0203 (0.0149)	0.0031 (0.0015)	2e-03 (5e-03)	1e-02 (1e-02)
	RUCC	Original	**Neg.**	**100**	0	31	-0.0747 (0.0309)		2e-04 (9e-04)	
		Alpha	**Neg.**	**100**	0	12	-0.0921 (0.0218)		5e-05 (1e-04)	
		Delta	**Neg.**	**100**	0	26	-0.0979 (0.0401)		7e-08 (3e-07)	
		Omicron	**Neg.**	**100**	0	63	-0.0847 (0.0933)		1e-04 (9e-04)	
