# Supplementary material for: Evolving patterns of COVID-19 mortality in US counties: A longitudinal study of healthcare, socioeconomic, and vaccination associations
Source: PLOS Glob Public Health. 2024 Sep 10;4(9):e0003590. doi: 10.1371/journal.pgph.0003590 (PMC11386416; doi:10.1371/journal.pgph.0003590)
Supplement: S2 Table — For each variable and variant period, the overall association, the week % of statistical significance, the coefficients, and the p-values are provided. Those with statistical significance during more than half of the weeks are highlighted in bold. (PDF) [file pgph.0003590.s012.pdf]

S2 Table. Summary of key results for OMHR with SVI Level III variables. For each variable and variant period, the overall association, the week % of statistical significance, the coefficients, and the p-values are provided. Those with statistical significance during more than half of the weeks are highlighted in bold.

	**Variable**	**Variant**	**Assoc.**	**Weeks significant**			**Coeffs. avg (std)**		**P-value* avg (std)**	
				(%)-	(%)+	Total	-	+	-	+
**SVI 1: Socioeconomic Status**	Below 150% Poverty	Original	**Pos.**	0	**65**	31		0.0054 (0.0024)		1e-02 (1e-02)
		Alpha	**Pos.**	0	**67**	12		0.0038 (0.0018)		7e-03 (1e-02)
		Delta	Insig.	0	31	26		0.0030 (0.0013)		0.02 (0.02)
		Omicron	**Pos.**	0	**54**	63		0.0065 (0.0077)		7e-03 (1e-02)
	Unemployed	Original	Insig.	6	0	31	-0.0067 (0.0028)		0.04 (0.01)	
		Alpha	Insig.	17	8	12	-0.0101 (0.0035)	0.0062 (nan)	0.01 (0.02)	0.02 (nan)
		Delta	Insig.	0	19	26		0.0044 (0.0012)		0.02 (0.01)
		Omicron	Insig.	3	19	63	-0.0053 (0.0012)	0.0027 (0.0011)	0.01 (0.02)	0.02 (0.01)
	Housing Cost Burden	Original	**Neg.**	**77**	0	31	-0.0075 (0.0032)		4e-03 (9e-03)	
		Alpha	Insig.	25	0	12	-0.0065 (0.0004)		4e-04 (1e-04)	
		Delta	Insig.	12	19	26	-0.0069 (0.0007)	0.0011 (0.0003)	1e-03 (7e-04)	0.02 (0.01)
		Omicron	Insig.	19	24	63	-0.0122 (0.0093)	0.0017 (0.0008)	5e-03 (1e-02)	1e-02 (1e-02)
	No High-School Diploma	Original	Insig.	0	35	31		0.0064 (0.0023)		0.02 (0.02)
		Alpha	Insig.	0	8	12		0.0051 (nan)		0.03 (nan)
		Delta	Insig.	0	35	26		0.0073 (0.0024)		9e-03 (9e-03)
		Omicron	Insig.	0	17	63		0.0115 (0.0076)		7e-03 (1e-02)
	No Health Insurance	Original	Insig.	16	6	31	-0.0059 (0.0014)	0.0031 (0.0002)	2e-03 (2e-03)	0.02 (0.02)
		Alpha	Insig.	17	17	12	-0.0042 (0.0032)	0.0028 (0.0002)	0.02 (0.03)	0.03 (0.01)
		Delta	Insig.	0	12	26		0.0074 (0.0022)		9e-03 (1e-02)
		Omicron	Insig.	16	8	63	-0.0014 (0.0005)	0.0047 (0.0047)	0.01 (0.01)	0.02 (0.01)
**SVI 2: Household Characteristics**	Aged 65 & Older	Original	**Pos.**	0	**87**	31		0.0091 (0.0032)		5e-03 (9e-03)
		Alpha	Insig.	25	0	12	-0.0048 (0.0008)		0.02 (0.02)	
		Delta	Insig.	0	0	26				
		Omicron	Insig.	30	8	63	-0.0044 (0.0043)	0.0071 (0.0090)	9e-03 (1e-02)	0.02 (0.01)
	Aged 17 & Younger	Original	**Pos.**	0	**61**	31		0.0141 (0.0063)		8e-03 (2e-02)
		Alpha	Insig.	0	33	12		0.0057 (0.0004)		0.02 (0.01)
		Delta	Insig.	4	0	26	-0.0086 (nan)		0.03 (nan)	
		Omicron	Insig.	13	2	63	-0.0043 (0.0036)	0.0428 (nan)	0.03 (0.01)	3e-03 (nan)
	Civilian with a Disability	Original	Insig.	35	0	31	-0.0057 (0.0017)		0.02 (0.02)	
		Alpha	**Pos.**	0	**50**	12		0.0064 (0.0028)		8e-03 (1e-02)
		Delta	Insig.	0	31	26		0.0042 (0.0032)		0.02 (0.02)
		Omicron	**Pos.**	0	**51**	63		0.0101 (0.0125)		1e-02 (1e-02)
	Single-Parent Households	Original	Insig.	0	35	31		0.0135 (0.0022)		9e-03 (1e-02)
		Alpha	Insig.	0	33	12		0.0105 (0.0028)		0.02 (0.01)
		Delta	**Pos.**	0	**50**	26		0.0084 (0.0053)		1e-02 (2e-02)
		Omicron	**Pos.**	0	**59**	63		0.0115 (0.0159)		0.01 (0.01)
	English Language Proficiency	Original	Insig.	45	0	31	-0.0087 (0.0026)		0.01 (0.01)	
		Alpha	**Neg.**	**58**	0	12	-0.0070 (0.0022)		9e-03 (7e-03)	
		Delta	**Neg.**	**54**	0	26	-0.0127 (0.0088)		6e-03 (1e-02)	
		Omicron	Insig.	27	0	63	-0.0217 (0.0184)		8e-03 (1e-02)	
**SVI 3**	Racial & Ethnic Minority Status	Original	Mixed	29	26	31	-0.0018 (0.0005)	0.0015 (0.0003)	4e-03 (7e-03)	5e-03 (6e-03)
		Alpha	Insig.	0	33	12		0.0016 (0.0007)		0.02 (0.02)
		Delta	Insig.	23	15	26	-0.0016 (0.0014)	0.0018 (0.0008)	0.01 (0.02)	0.02 (0.02)
		Omicron	Insig.	49	3	63	-0.0013 (0.0012)	0.0020 (0.0003)	4e-03 (9e-03)	0.01 (0.02)

Continued: Summary of results for OMHR - Level III

	Variable	Variant	Assoc.	Weeks significant			Coeffs. avg (std)		P-value* avg (std)	
				(%)-	(%)+	Total	-	+	-	+
SVI 4: Housing Type & Transportation	Multi-Unit Structures	Original	Insig.	3	10	31	-0.0042 (nan)	0.0031 (0.0005)	0.02 (nan)	0.04 (0.00)
		Alpha	Insig.	0	0	12				
		Delta	Insig.	12	0	26	-0.0015 (0.0004)		0.02 (0.01)	
		Omicron	Insig.	25	6	63	-0.0017 (0.0011)	0.0028 (0.0008)	0.01 (0.01)	4e-03 (5e-03)
	Mobile Homes	Original	**Neg.**	**55**	0	31	-0.0033 (0.0012)		0.01 (0.01)	
		Alpha	Insig.	17	0	12	-0.0013 (0.0004)		0.04 (0.01)	
		Delta	Insig.	0	27	26		0.0032 (0.0010)		0.01 (0.02)
		Omicron	Insig.	41	5	63	-0.0022 (0.0020)	0.0010 (0.0002)	8e-03 (1e-02)	0.03 (0.02)
	Crowding	Original	**Neg.**	**52**	0	31	-0.0148 (0.0057)		0.01 (0.01)	
		Alpha	Insig.	42	0	12	-0.0110 (0.0027)		2e-03 (2e-03)	
		Delta	Insig.	35	0	26	-0.0112 (0.0065)		4e-03 (6e-03)	
		Omicron	**Neg.**	**63**	0	63	-0.0100 (0.0088)		9e-03 (1e-02)	
	No Vehicle	Original	Insig.	42	0	31	-0.0082 (0.0034)		6e-03 (7e-03)	
		Alpha	**Neg.**	**92**	0	12	-0.0065 (0.0026)		5e-03 (9e-03)	
		Delta	**Neg.**	**69**	0	26	-0.0084 (0.0054)		3e-03 (5e-03)	
		Omicron	**Neg.**	**60**	0	63	-0.0136 (0.0165)		6e-03 (1e-02)	
	Group Quarters	Original	**Pos.**	3	**68**	31	-0.0051 (nan)	0.0057 (0.0021)	0.02 (nan)	0.01 (0.02)
		Alpha	Insig.	25	0	12	-0.0035 (0.0007)		0.02 (0.01)	
		Delta	**Neg.**	**50**	0	26	-0.0044 (0.0018)		9e-03 (1e-02)	
		Omicron	**Neg.**	**54**	0	63	-0.0030 (0.0029)		0.01 (0.01)	
non-SVI variables	Beds	Original	**Neg.**	**100**	0	31	-0.0347 (0.0085)		3e-22 (1e-21)	
		Alpha	**Neg.**	**100**	0	12	-0.0143 (0.0048)		4e-14 (1e-13)	
		Delta	**Neg.**	**100**	0	26	-0.0229 (0.0170)		5e-11 (2e-10)	
		Omicron	**Neg.**	**100**	0	63	-0.0200 (0.0264)		6e-08 (3e-07)	
	Vaccination Coverage	Original	Insig.	9	9	11	-0.0069 (nan)	0.0190 (nan)	0.02 (nan)	0.02 (nan)
		Alpha	Insig.	0	42	12		0.0019 (0.0007)		8e-03 (1e-02)
		Delta	**Neg.**	**62**	8	26	-0.0023 (0.0011)	0.0004 (0.0000)	3e-03 (1e-02)	0.01 (0.01)
		Omicron	Insig.	17	33	63	-0.0064 (0.0041)	0.0007 (0.0002)	5e-03 (8e-03)	8e-03 (1e-02)
	RUCC	Original	Insig.	42	10	31	-0.0096 (0.0023)	0.0093 (0.0023)	0.01 (0.01)	1e-02 (9e-03)
		Alpha	**Neg.**	**92**	0	12	-0.0105 (0.0032)		7e-03 (2e-02)	
		Delta	**Neg.**	**85**	0	26	-0.0099 (0.0043)		2e-03 (6e-03)	
		Omicron	**Neg.**	**84**	0	63	-0.0096 (0.0080)		3e-03 (9e-03)	
