# Supplementary material for: Evolving patterns of COVID-19 mortality in US counties: A longitudinal study of healthcare, socioeconomic, and vaccination associations
Source: PLOS Glob Public Health. 2024 Sep 10;4(9):e0003590. doi: 10.1371/journal.pgph.0003590 (PMC11386416; doi:10.1371/journal.pgph.0003590)
Supplement: S3 Table — (PDF) [file pgph.0003590.s013.pdf]

S3 Table. R^2 values for each SVI level of analyses.

		CFR R^2 (%)			OMHR R^2 (%)		
	**Time Period**	**range**	**mean**	**std**	**range**	**mean**	**std**
Level I	Original	[2-11]	5.7	2.6	[6-11]	8.8	1.3
	Alpha	[7-9]	7.6	0.7	[5-7]	5.8	0.7
	Delta	[7-26]	14.4	6.3	[7-15]	10.6	2.4
	Omicron	[2-14]	7.4	2.6	[2-11]	6.4	2.2
Level II	Original	[4-13]	7.4	2.6	[6-12]	9.5	1.5
	Alpha	[8-11]	9.5	1.0	[6-8]	6.7	0.7
	Delta	[8-27]	16.2	6.2	[8-16]	11.7	2.6
	Omicron	[5-19]	10.9	3.0	[4-13]	7.8	2.6
Level III	Original	[6-15]	9.8	2.4	[8-16]	11.1	1.9
	Alpha	[8-12]	10.4	1.2	[6-8]	7.7	0.7
	Delta	[9-29]	16.6	6.9	[8-19]	12.9	3.7
	Omicron	[6-19]	12.3	3.1	[5-15]	9.4	2.8
