# Supplementary material for: Evolving patterns of COVID-19 mortality in US counties: A longitudinal study of healthcare, socioeconomic, and vaccination associations
Source: PLOS Glob Public Health. 2024 Sep 10;4(9):e0003590. doi: 10.1371/journal.pgph.0003590 (PMC11386416; doi:10.1371/journal.pgph.0003590)
Supplement: S4 Table — The associations between SVI theme variables and CFR are presented in Fig 7. For each variable and variant period, the overall association, the week % of statistical significance, the coefficients, and the p-values are provided. (PDF) [file pgph.0003590.s014.pdf]

S4 Table. Summary of key results for CFR with non-SVI explanatory variables in the Level II analysis. The associations between SVI theme variables and CFR are presented in Figure ??. For each variable and variant period, the overall association, the week % of statistical significance, the coefficients, and the p-values are provided.

**Variable**	**Variant**	**Assoc.**	**Weeks significant**			**Coeffs. avg (std)**		**P-value* avg (std)**	
			**(%)-**	**(%)+**	**Total**	-	+	-	+
Beds	Original	Insig.	10	6	31	-0.0191 (0.0031)	0.0379 (0.0017)	0.02 (0.01)	4e-03 (4e-03)
	Alpha	**Neg.**	**83**	0	12	-0.0261 (0.0089)		3e-03 (6e-03)	
	Delta	**Neg.**	**96**	0	26	-0.0210 (0.0085)		3e-03 (5e-03)	
	Omicron	**Neg.**	**95**	0	63	-0.0186 (0.0190)		2e-03 (4e-03)	
Vaccination Coverage	Original	Insig.	45	0	11	-0.0670 (0.0681)		9e-03 (2e-02)	
	Alpha	Insig.	42	0	12	-0.0117 (0.0025)		0.01 (0.01)	
	Delta	**Neg.**	**73**	15	26	-0.0148 (0.0077)	0.0078 (0.0026)	2e-04 (1e-03)	9e-03 (1e-02)
	Omicron	Mixed	30	33	63	-0.0255 (0.0215)	0.0034 (0.0015)	7e-04 (3e-03)	1e-02 (1e-02)
65+ Percentage	Original	**Pos.**	0	**87**	31		0.0282 (0.0106)		4e-03 (1e-02)
	Alpha	Insig.	8	0	12	-0.0167 (nan)		0.03 (nan)	
	Delta	**Pos.**	0	**65**	26		0.0245 (0.0062)		4e-03 (1e-02)
	Omicron	**Pos.**	2	**56**	63	-0.0067 (nan)	0.0178 (0.0136)	0.01 (nan)	5e-03 (1e-02)
RUCC	Original	**Neg.**	**81**	0	31	-0.0550 (0.0263)		6e-03 (1e-02)	
	Alpha	**Neg.**	**100**	0	12	-0.0924 (0.0353)		2e-04 (5e-04)	
	Delta	**Neg.**	**100**	0	26	-0.1089 (0.0459)		2e-16 (7e-16)	
	Omicron	**Neg.**	**100**	0	63	-0.0858 (0.0661)		2e-07 (1e-06)	
