# Supplementary material for: Evolving patterns of COVID-19 mortality in US counties: A longitudinal study of healthcare, socioeconomic, and vaccination associations
Source: PLOS Glob Public Health. 2024 Sep 10;4(9):e0003590. doi: 10.1371/journal.pgph.0003590 (PMC11386416; doi:10.1371/journal.pgph.0003590)
Supplement: S5 Table — The associations between SVI theme variables and OMHR are presented in Fig 7. For each variable and variant period, the overall association, the week % of statistical significance, the coefficients, and the p-values are provided. (PDF) [file pgph.0003590.s015.pdf]

S5 Table. Summary of key results for OMHR with non-SVI explanatory variables in the Level II analysis. The associations between SVI theme variables and OMHR are presented in Figure **??**. For each variable and variant period, the overall association, the week % of statistical significance, the coefficients, and the p-values are provided.

**Variable**	**Variant**	**Assoc.**	**Weeks significant**			**Coeffs. avg (std)**		**P-value* avg (std)**	
			**(%)-**	**(%)+**	**Total**	-	+	-	+
Beds	Original	**Neg.**	**100**	0	31	-0.0337 (0.0093)		2e-21 (9e-21)	
	Alpha	**Neg.**	**100**	0	12	-0.0139 (0.0043)		1e-14 (3e-14)	
	Delta	**Neg.**	**100**	0	26	-0.0234 (0.0176)		3e-10 (1e-09)	
	Omicron	**Neg.**	**100**	0	63	-0.0202 (0.0269)		7e-09 (4e-08)	
Vaccination Coverage	Original	Insig.	27	0	11	-0.6836 (1.1718)		0.03 (0.01)	
	Alpha	**Pos.**	0	**67**	12		0.0016 (0.0006)		4e-03 (8e-03)
	Delta	**Neg.**	**58**	27	26	-0.0032 (0.0015)	0.0006 (0.0003)	5e-05 (2e-04)	6e-03 (6e-03)
	Omicron	Mixed	25	27	63	-0.0071 (0.0060)	0.0007 (0.0002)	5e-03 (1e-02)	5e-03 (9e-03)
65+ Percentage	Original	Insig.	10	3	31	-0.0033 (0.0012)	0.0022 (nan)	0.03 (0.01)	0.04 (nan)
	Alpha	**Neg.**	**50**	0	12	-0.0033 (0.0013)		0.01 (0.01)	
	Delta	Insig.	0	8	26		0.0019 (0.0005)		0.02 (0.01)
	Omicron	Insig.	11	27	63	-0.0020 (0.0010)	0.0019 (0.0006)	1e-02 (1e-02)	0.02 (0.02)
RUCC	Original	**Pos.**	6	**55**	31	-0.0057 (0.0003)	0.0157 (0.0059)	0.02 (0.01)	3e-03 (9e-03)
	Alpha	**Neg.**	**67**	0	12	-0.0090 (0.0032)		1e-03 (4e-03)	
	Delta	**Neg.**	**62**	0	26	-0.0082 (0.0051)		3e-03 (7e-03)	
	Omicron	**Neg.**	**70**	0	63	-0.0080 (0.0028)		4e-04 (2e-03)	
